# A pre-post pilot study of a brief, web-based intervention to engage disadvantaged smokers into cessation treatment

**DOI:** 10.1186/s13722-015-0026-5

**Published:** 2015-02-01

**Authors:** Mary F Brunette, William Gunn, Hilary Alvarez, Patricia C Finn, Pamela Geiger, Joelle C Ferron, Gregory J McHugo

**Affiliations:** Geisel School of Medicine at Dartmouth, Department of Psychiatry, Psychiatric Research Center, 105 Pleasant St, Concord, NH 03301 USA; Concord Hospital Family Health Center, Concord, USA

**Keywords:** Tobacco cessation, Disadvantaged populations, Technology, Motivation

## Abstract

**Background:**

People with low education and/or income are more likely to smoke, less likely to quit, and experience disparately poor health outcomes compared to those with education and income advantage. Cost-effective strategies are needed to inform and engage this group into effective cessation treatments. We developed a novel, web-based, motivational, decision-support system that was designed to engage disadvantaged smokers into tobacco cessation treatment. We piloted the system among smokers in a primary care safety net clinic.

**Methods:**

Thirty-nine eligible subjects were assessed at baseline and used the decision-support system; 38 were assessed 2 months later. Chi-square or Fisher’s exact tests were used to assess whether participants who used the program were more likely to use cessation treatment than a randomly selected group of 60 clinic patients.

**Results:**

Thirty-nine percent of smokers initiated cessation treatment after using the decision-support system, compared to 3 percent of the comparison group (Fisher’s exact = 21.2; p = 0.000). Over 10 percent achieved continuous abstinence over the 2-month follow-up. Users were satisfied with the program – 100 percent stated they would recommend it to a friend.

**Conclusions:**

Our data indicate that this web-based, motivational, decision-support system is feasible, satisfactory, and promising in its ability to engage smokers into cessation treatment in a primary care safety net clinic. Further evaluation research is warranted.

## Introduction

Cigarette smoking remains the leading preventable cause of morbidity and mortality in the United States, causing almost 500,000 deaths each year due to cardiovascular diseases, lung diseases, and many kinds of cancers [1]. Since 1950, the prevalence of smoking has declined from over 50 percent to just under 20 percent of the general population [2,3], but the rates of smoking among those with low levels of education have remained high in men and have increased in women [2]. Currently, disadvantaged individuals (with lower educational attainment, unemployment, or living in poverty) are much more likely to smoke than others [4-10], and they experience early morbidity and mortality due to smoking-related diseases [6]. Many in this group access health care in safety net clinics designated to provide primary care, including maternity and child health services, to underserved populations.

Fortunately, quitting at any age reduces morbidity and increases life expectancy [11]. A large body of research, summarized with meta-analyses in a 2008 report from the U.S. Department of Health and Human Services, shows that medication and behavioral treatment improve the likelihood of cessation [12]. Although disadvantaged smokers may not respond as robustly to evidence-based cessation treatment as advantaged smokers [13], such treatment significantly improves their cessation outcomes [14-17]. A key strategy to improve health outcomes in this group is therefore to facilitate use of cessation treatments [18-21].

Disadvantaged smokers want to quit and attempt to do so frequently [8,10,22-24]. But lack of knowledge, misinformation, and negative attitudes about cessation treatment impede their use [8,14,25-30] and their ability to facilitate abstinence. Several models of clinical intervention, such as education, decision support, and motivational counseling, increase patients’ knowledge of and interest in behavior change [28,31-35]. Unfortunately, these motivation-stage (precontemplation or contemplation) interventions are not broadly available because of competing demands and the lack of adequately trained staff in the busy clinics where these smokers obtain care.

### Using technology can improve access to and quality of interventions

Internet websites show promise for engaging large numbers of people into important health decisions, such as quitting smoking, thus improving health outcomes with little clinician time and cost [36]. Smoking cessation websites are effective [37] and available on the internet [38]. Unfortunately, when we assessed four of these easily accessible websites, including high-quality sites, we found that disadvantaged smokers with low education, low computer experience, and mental illness could not use them [39]. We therefore developed an easy-to-use, web-based, motivational, decision-support system called *Let’s Talk About Smoking* (described below). The purpose of this pilot study was to assess whether this web-based, motivational, decision-support system could engage smokers who were not motivated to use treatment in a primary care “safety net” clinic that serves disadvantaged people.

## Materials and methods

### Overview

After baseline assessments, eligible, consenting smokers used the decision-support system on a computer in the safety net clinic. After 2 months, we assessed subjects for past 2-month use of cessation treatment (main outcome) as well as smoking characteristics and abstinence. We determined the rate of cessation treatment utilization in a clinic comparison group by reviewing randomly selected charts of smokers seen for a primary care visit in the clinic during the 6-month study period. The study protocol was reviewed and approved by the Dartmouth College Committee for the Protection of Human Subjects. All procedures followed guideline from the Declaration of Helsinki.

### Intervention

*Let’s Talk About Smoking* is a website that was designed to motivate smokers to quit by using evidence-based cessation treatment. It was developed with extensive testing among disadvantaged smokers to inform its design [40,41] and was tailored for use in primary care, based on focus group input from primary care patients. The program delivers motivational content and decision support for cessation treatment (e.g., behavioral support, in which treatment is recommended and the choice of one treatment option over another may be influenced by patient characteristics) [42]. The program delivers a choice of culturally diverse patient program guides, five interactive educational modules, and video recorded patient quit stories. Through the program, users evaluate the financial cost of their smoking, explore the impact of smoking on their health, create a personal list of pros and cons, and watch quit stories of people like them who used cessation treatment to quit (including primary care patients). Part of the program was tailored for pregnant women, who have different reasons for smoking and quitting, and fewer evidence-based treatment options. This content included information about the health impact of smoking on pregnancy and children, as well as video recorded quit stories of other pregnant women, viewed only if the user indicated she was pregnant. Users viewed information about evidence-based cessation treatments, with the amount of information tailored to their level of interest. Based on the Theory of Planned Behavior [43], the program targets treatment attitudes, social norms, and perceived behavioral control regarding cessation treatment. The simple tailored design, use of plain language, and text-to-speech functionality makes this program easy to use by smokers with low education, cognitive impairments, or low computer experience [40]. Use of the program takes between 45 and 90 minutes, depending upon how much time the user chooses to spend and how much information he or she chooses to view. Previous work conducted in mental health clinics with disadvantaged smokers demonstrated that users with cognitive deficits and lower education take more time viewing the program but still complete it with similar outcomes [44].

### Setting

The study took place in a primary care clinic designated to provide care to underserved citizens, including people without insurance, people living in poverty, and refugees. The clinic has over 30 doctors, including doctors in a family practice residency training program. Patients of all doctors were invited to participate in the study.

### Study participants

Safety net clinic patients were eligible if they were: age 18–70 years, smoking four cigarettes or more per day, fluent in English, and willing to give informed consent. Based on chart review, patients were excluded if they: had used evidence-based smoking cessation treatment within the past 2 months (indicating the patient was already motivated or engaged in treatment) or had current untreated alcohol or drug dependence (because dependence can impede cessation treatment and requires different treatment approaches [45,46]). Pregnant women were included and given a version of the program that was tailored for them, with information about the health impact of smoking on pregnancy and children and video recorded quit stories of other pregnant women. Since this intervention was designed to increase motivation for cessation, intention to quit smoking was not required. All subjects were patients at the primary care safety net clinic described above, which has a federal designation as a clinic that provides care to underserved uninsured people and Medicaid recipients.

### Participant flow

During a 4-month period, clinic staff gave information about the study to all patients who smoked at the time medical staff checked patients’ smoking status. Patients were told the purpose of the study was to try a website for smokers. If a patient indicated they were interested, their name was given to research staff, who then called them to explain the study. Clinic staff referred 124 participants to research staff, who reached and briefly screened 104 participants over the phone. Eighteen participants were not eligible and 43 had scheduling difficulties or chose not to participate for other reasons. Among the referred group, 45 provided informed consent to participate in the study. After baseline assessment, 41 were found to be eligible for the study, 39 used the website decision-support system, and 38 (96.7%) were assessed 2 months later. We reported outcomes on the 38 whom we were able to assess for outcomes.

### Study design

After providing informed consent, subjects were assessed at baseline for: demographics, diagnoses, smoking characteristics, use of cessation treatment in the past 2 months, mental health distress, and reading comprehension. Eligible participants then used the computer program while being observed by research staff. After finishing the program, computerized questionnaires assessed participants for satisfaction with the decision-support system as well as intention to quit smoking and use cessation treatment. Research staff then offered participants a referral sheet that listed treatment options at the clinic as well as quitline information. Subjects could easily access cessation medications via clinic doctors, who attended a brief training at the beginning of the study period on evidence-based cessation pharmacotherapy. Behavioral treatment was available at the site (six sessions of standard in-person counseling) and also via the state-sponsored telephone quitline (three phone counseling sessions). Two months later, research staff met in person with subjects to assess them for smoking characteristics, self-reported use of cessation treatment, and other quit behaviors in the past 2 months. Chart review and clinician report were obtained to confirm self-reported use of cessation treatment.

### Measures

Research staff obtained baseline demographics and history with a structured interview. Medical record review determined current diagnoses of health conditions, mental illnesses, and substance dependence. Research staff conducted baseline assessments, including the Colorado Symptom Index (CSI) to assess mental health symptoms and distress [47], the Multidimensional Scale of Perceived Social Support (MSPSS) [48] to assess social support, and the Wide Range Achievement Test to assess reading comprehension [49].

Smoking characteristics and use of cessation treatment were assessed at baseline and at 2 months. The research staff assessed the main outcome and use of smoking cessation treatment, with a self-report checklist that included starting any cessation medication (prescribed or over-the-counter) and attending any behavioral treatment (in-person counseling and/or telephone quitline) [50]. Receipt of prescription cessation medication and use of clinic-based behavioral cessation treatment was confirmed by clinicians. (We chose treatment initiation behavior as our main outcome, rather than intention, beliefs, or attitudes, because treatment initiation is a rigorous measure of motivation to use treatment [51].) Quit attempts and days of abstinence over the past 2 months were also assessed by structured interview. Amount of smoking over the past 2 months was assessed with structured interview quantity/frequency questions. The Fagerstrom Test for Nicotine Dependence, a five-item measure, assessed nicotine dependence [52,53]. We measured satisfaction with the decision-support system directly after its use with an adapted, computerized, 15-item, semiqualitative questionnaire [54].

### Comparison group

To assess the rate of use of cessation treatment in a comparison group, we randomly selected 60 records of adult smokers who had a primary care doctor visit during the same time period. Four of the selected records belonged to people who signed up for the study and were replaced by four new randomly selected records. Research staff reviewed these records for evidence of prescription cessation medication and use of cessation counseling. Clinic staff obtained self-report confirmation of actual use of cessation treatment from those whose records indicated treatment was prescribed or delivered. No other research measures were obtained from the comparison group.

### Statistical analysis

We used summary statistics to describe the study participants, their flow through the protocol, their satisfaction with the decision-support system, and the main outcome, treatment initiation. The main outcome of interest concerns the proportion of participants who initiated cessation treatment in the 2 months following their use of the decision-support system. Chi-square or Fisher’s exact tests were used to test the observed proportion initiating treatment against the observed rate of treatment initiation at the clinic. In planning this analysis, we assessed power with a one-tailed test against .10 at p < .05 with n = 20. We found we had 80 percent power to detect a proportion of .32 or greater in the intervention group, indicating that this study had adequate power for a pilot test of this intervention. We also explored the relationship between nonpregnant participant characteristics and outcomes with logistic regressions. Because the pregnant group was small (n = 9), we did not conduct statistical tests to assess these relationships.

## Results and discussion

Characteristics of the 38 participants who completed all assessments are shown in Table 1. Nonpregnant participants were middle-aged, mostly white smokers with multiple chronic medical conditions who smoked on average about one pack a day. Most also had psychiatric disorders. They were disadvantaged: most were unemployed and had low education level, reading level, and income. Pregnant participants were mostly young, employed white women with chronic health conditions who smoked an average of about 11 cigarettes a day. Over half had psychiatric diagnoses. They also were disadvantaged in terms of education and income. Knowledge about smoking was good: all answered three questions about the health effects of smoking correctly (causes lung disease, heart disease, cancer, and wrinkles). About 10 percent of the group was misinformed about each type of cessation medication.

**Table 1:** **Characteristics of study participants (N = 38)**

	**Nonpregnant**		**Pregnant**		**All**	
	**n = 29**		**n = 9**		**N = 38**	
Mean (sd) age	46.0	(14.1)	26.3	(5.4)	41.7	(15.1)
Number (%) male	14	(48)	0	(0)	14	(37)
Number (%) White	29	(100)	8	(89)	37	(97)
Number (%) Black or Native American	0	(0)	0	(0)	0	(0)
Number (%) non-Hispanic	29	(100)	9	(100)	38	(100)
Mean (sd) years education	11.9	(2.3)	11.1	(2.3)	11.7	(2.3)
Mean (sd) WRAT reading score	41.0	(7.8)	35	(8.1)	39.4	(8.8)
Number (%) married or living with partner	10	(34)	8	(89)	18	(47)
Number (%) employed	8	(28)	8	(89)	16	(42)
Mean (sd) monthly income (dollars)	1526.5	(1055.2)	1532.1	(914.3)	1527.8	(1014.9)
Number (range) with chronic medical condition	27.0	(93)	7	(78)	34	(89)
Median (range) chronic medical conditions	3.5	(0–7)	1	(0–2)	3	(0–7)
Number (%) with psychiatric diagnosis	23.0	(79)	5	(56)	28	(74)
Mean (sd) CSI score (0–56)	18.1	(9.2)	16.8	(5.6)	17.8	(8.5)
Mean (sd) Fagerstrom score	5.7	(1.8)	3.2	(2.5)	5.1	(2.2)
Mean (sd) cigarettes/day	19.6	(8.3)	10.8	(8.6)	17.6	(9.0)
Number (%) used a computer > 5 times	26	(90)	9	(100)	35	(92)
Number (%) used Internet for health information	19.0	(66)	7	(78)	26	(68)

WRAT = Wide Range Achievement Test for reading ability;

CSI = Colorado Symptom Index for mental health distress.

Satisfaction with the decision-support system was high. All users who started the program completed it. All users rated the way the information was presented as good or excellent, 93 percent were somewhat or very satisfied with the program, and 100 percent said they would recommend the program to a friend.

Use of cessation treatment and other quit behaviors during the 2 months following use of the motivational decision-support system are shown in Table 2, reported by pregnancy status. Many participants tried to quit after using *Let’s Talk About Smoking*. Over one-third of the 39 participants (35.9%) reported they had used cessation treatment. Five people used nicotine replacement therapy, and one person used counseling not confirmed by chart review (these presumably were accessed outside of the clinic). Almost one-third initiated evidence-based cessation treatment that was confirmed by chart review, compared to 3 percent of the chart review clinic comparison group (Fishers exact = 21.2; p = 0.000). Rates of cessation treatment use were higher among nonpregnant smokers than among pregnant smokers.

**Table 2:** **Use of cessation treatment and smoking outcomes over 2 months**

	**Nonpregnant**		**Pregnant**		**All**	
	**n = 29**		**n = 9**		**N = 38**	
**Cessation treatment and attempts**						
Number (%) used any treatment	14	(48)	1	(11)	15	(39)
Number (%) used chart-confirmed treatment	10	(34)	1	(11)	11	(29)
Number (%) used medication only*	4	(14)	0	(0)	4	(11)
Number (%) used NRT only (OTC)*	3	(10)	1	(11)	4	(11)
Number (%) used counseling only	1	(3)	0	(0)	1	(3)
Number (%) used both med/NRT* and counseling	5	(17)	0	(0)	5	(13)
Number (%) tried 'cold turkey'	12	(41)	4	(44)	16	(42)
Number (%) tried to quit in past 2 months	21	(72)	6	(67)	27	(71)
**Smoking outcomes**						
Number (%) achieved ≥1 day abstinence	13	(45)	5	(56)	18	(47)
Number (%) achieved ≥ 7 days abstinence	6	(21)	1	(11)	7	(18)
Mean (sd) days abstinence among quitters	10.9	(11.9)	5	(7.3)	9.3	(11.0)
Number (%) achieved continuous abstinence	3	(10)	1	(11)	4	(11)
Mean (sd) number cigarettes/day in smokers	14.3	(9.7)	9	(6.6)	12.8	(9.0)

NRT = nicotine replacement therapy; OTC = over the counter.

*Medication and nicotine replacement therapy were not recommended for pregnant women.

Among nonpregnant participants, higher levels of education and lower number of cigarettes smoked at baseline marginally predicted use of cessation treatment after using the decision-support system (p = 0.06). Age, gender, Fagerstrom Nicotine Dependence score, number of cigarettes smoked per day, level of reading comprehension, amount of mental health distress (CSI score) and level of social support (MSPSS score) did not predict using cessation treatment.

Cessation outcomes are also shown in Table 2. Eighteen participants who used the decision-support system (47%) became abstinent for at least a day, seven (18.4%) achieved abstinence for seven or more days, and four (10.5%) became continuously abstinent. Among nonpregnant participants, days of abstinence trended higher among those who used any evidence-based cessation treatment compared to those who did not (mean days abstinence 11.0 ± 14.3 vs. 1.7 ± 2.8; t = −2.04, df = 9.4, p = .07). Achieving abstinence was predicted by a lower number of cigarettes smoked per day at baseline (p = .05), but not by age, reading capability, Fagerstrom score, number of cigarettes smoked per day, level of mental health distress (CSI score), and level of social support (MSPSS score). Although accessing evidence-based treatment predicted days of abstinence (p < .04), accessing free medication (vs. use of insurance or self pay) was not associated with days of abstinence. Among those who continued to smoke, the mean number of cigarettes smoked per day significantly decreased from a mean of 18.1 ± 9.1 cigarettes to 12.8 ± 8.9 cigarettes (t = 3.4, df = 36, p = .002).

The single-session, web-based, motivational, decision-support system was feasible and acceptable to safety net clinic primary care patients. All participants who started using the program completed it, and satisfaction with the program was high. Our research also suggests that the decision-support system may be a promising strategy to engage users in cessation treatment: most tried to quit and over one-third of users went on to initiate cessation treatment, compared to only 3 percent of the clinic comparison group. Further, 11 percent of the study group had achieved continuous abstinence when assessed 2 months after using the intervention, and abstinence was associated with using cessation treatment in nonpregnant participants. These findings are similar to previous research on a kiosk computer website decision-support system studied in underserved Latino communities [55], suggesting that such websites are a promising and potentially low-cost method for engaging disadvantaged smokers into effective cessation treatments.

Technology (computer, phone, or tablet) holds great promise for delivering decision support and interventions of varying duration that can extend and enhance the treatment delivered in under-resourced primary care and addiction treatment settings [56,57]. Notably, over three-quarters of our study group had used the Internet to obtain health information, and satisfaction with the program was high, suggesting that the Internet is a highly acceptable medium for this population. Further, technology can enable delivery of consistent, high-fidelity intervention to users every time. In contrast to phone interventions, computerized interventions use an easy-to-read large screen to provide assessments, provide information, and teach skills using multiple media formats in clinic or home settings. Cell phones can also deliver mobile interventions in the patient’s environment and have been shown to be helpful for people who smoke [58], although their smaller screens may limit the type of intervention being delivered. We designed the intervention studied here, *Let’s Talk About Smoking*, with multiple features that enhanced usability for disadvantaged populations who may have low education, minimal computer experience, and cognitive difficulties [40]. Additionally, for this study we implemented the intervention with a clinic facilitation model (clinic referral and in-clinic website use) that provided structure during the brief intervention to patients who were not yet motivated to address their tobacco use with cessation treatment.

Disadvantaged pregnant smokers are a particularly challenging population to help with smoking cessation. In this clinic, 45 percent of the 157 pregnant women seen in the past year reported they had been smoking when they learned they were pregnant, and a large majority continued smoking during their pregnancy. After using our web-based intervention, abstinence outcomes among our small sample of disadvantaged pregnant participants were the same as for the nonpregnant participants, but lower than rates found in other studies of brief motivational interventions for pregnant smokers [59]. Although the pregnant women received information about the impact of smoking on their pregnancy and the fetus, as well as standard motivational interviewing and decision-support content, the program did not have a big effect. The website may need to be tailored further for pregnant women, and clinic resources may need enhancement to implement behavioral cessation treatments tailored to pregnant women.

This pilot study was limited by several factors, including the small number of subjects (and very small number who were pregnant). However, we did have the power to detect a difference between the rate of treatment use among smokers using the decision-support system and comparison rates; thus, our significant findings are meaningful despite the small number enrolled in the study. Further, our rate of follow-up assessments was excellent. Secondly, we pilot-tested this intervention in a group of participants who were willing to participate in research. It is possible that the outcomes in this small group may not generalize to the entire clinic. Further, whether the outcomes we found at a safety net clinic in this small, primarily white, urban community would be similar in communities with different characteristics is not known. Although the generalizability of our findings needs to be evaluated with further study, the outcomes we found here are similar to outcomes we found and previously reported among disadvantaged African American smokers in urban Chicago mental health clinics [50]. Third, we did not obtain biologic verification of abstinence, a secondary outcome. We did verify our main outcome, cessation treatment, with clinic records, a significant strength of the study. We did not obtain demographic characteristics of the randomly selected control group so are unable to know if they are different from study participants who received the intervention. Finally, study participants could access free nicotine replacement therapy, the absence of which may have limited the rate of engagement into treatment among the comparison group, although all patients had access to other cessation medication samples and the telephone quitline counseling.

### Conclusions

Data from this pilot test of a web-based, motivational, decision-support system are promising and suggest that it may be a low-cost strategy to facilitate use of cessation treatment, a key pathway to abstinence among smokers in safety net clinics. Further research is needed to assess the efficacy of this approach within randomized controlled trials in diverse settings with racially heterogeneous groups of smokers, as well as to evaluate strategies to implement technology-delivered interventions in health care and other settings.
